# Construct Validation of the Dietary Inflammatory Index (DII) among Young College-Aged Women

**DOI:** 10.3390/nu15214553

**Published:** 2023-10-27

**Authors:** Deniz Azarmanesh, Jessica Pearlman, Elena T. Carbone, Janie C. DiNatale, Elizabeth R. Bertone-Johnson

**Affiliations:** 1 Department of Human Nutrition and Hospitality Management, The University of Alabama, Tuscaloosa, AL 35487, USA; jcdinatale@crimson.ua.edu; 2 Institute for Social Science Research, University of Massachusetts Amherst, Amherst, MA 01003, USA; jpearlman@issr.umass.edu; 3 Department of Nutrition and Commonwealth Honors College, University of Massachusetts Amherst, Amherst, MA 01003, USA; ecarbone@umass.edu; 4 Department of Health Promotion and Policy, University of Massachusetts Amherst, Amherst, MA 01003, USA; ebertone@umass.edu

**Keywords:** Dietary Inflammatory Index, DII, construct validation, inflammation, cytokines, young women

## Abstract

The Dietary Inflammatory Index (DII) is designed to assess the inflammatory potential of the diet. While previous research has utilized DII among college-aged women, no study to date has validated it in this population. We conducted a construct validation of DII among 393 healthy women aged 18–31 years against a robust panel of 14 inflammatory biomarkers, including CRP, IL-1β, IL-4, IL-6, IL-10, and TNF-α, which were used in the development of DII. Three linear regression models were constructed: (1) an age-adjusted model, (2) the most parsimonious model based on likelihood ratio tests, and (3) a fully adjusted model for age, race, body mass index, waist circumference, physical activity, smoking status, and nonsteroidal anti-inflammatory drug use. DII was derived from the Harvard food frequency questionnaire and categorized into quartiles. Consistent with our hypothesis, DII was negatively and significantly associated with back-transformed IL-10 levels, confirming that a more pro-inflammatory diet was associated with lower levels of an anti-inflammatory cytokine (Model 3: Q4 vs. Q1 β = 0.62; 95% CI: 0.42, 0.93; *p*-trend = 0.04). While validated in other populations, DII may not be a suitable tool for assessing the inflammatory potential of the diet among college-aged women.

## 1. Introduction

Inflammation is a normal function of the body’s immune system that works against pathogens and injuries [1,2]. A prolonged inflammatory response, however, is damaging to body tissues and organs. Anti-inflammatory cytokines (e.g., IL-10) are part of a negative feedback system that help inhibit pro-inflammatory signaling [1]. When these regulatory processes fail, it can lead to the development of chronic systemic inflammation [1]. Systemic inflammation is characterized by increased pro-inflammatory biomarkers in circulation, including interleukin (IL)-6, C-Reactive Protein (CRP), and tumor necrosis factor alpha (TNF-α). This inflammatory response occurs due to non-modifiable factors (e.g., hormones, aging), and modifiable and environmental factors, such as diet [3,4,5,6,7]. Mechanistic studies have shown that diet modulates inflammation through the impact of dietary components on oxidative stress, nuclear factor kappa B (NF-κB) activation, the activity of the sympathetic nervous system, and upregulation in the production of pro-inflammatory cytokines [8]. Further mechanistic studies have linked diet to systemic inflammation through the dietary activation of the immune system, as well as lipoprotein and adipose tissue dysfunction [9,10,11]. Chronic systemic inflammation can lead to various health problems, such as cancer, arthritis, cardiovascular disease, diabetes, and neurological diseases [12]. Previous research has shown that Western dietary patterns, characterized as being highly processed and containing high-fat dairy and red meat, refined grains, fried foods, and sweets, are associated with chronic systemic inflammation and a higher presence of circulatory pro-inflammatory biomarkers (e.g., CRP, TNF-α, IL-6). However, following healthy dietary patterns, such as the Mediterranean diet, characterized as being high in whole grains, fruits, vegetables, and lean meats, has been linked to higher levels of anti-inflammatory (e.g., IL-10) biomarkers [13,14].

Researchers to date have mainly focused on examining the association of single nutrients with inflammation or chronic diseases, which does not consider the interactions of nutrients when consumed together [6]. Additionally, many dietary indices often base their assessment on previous recommendations of intake [14]. For example, the Healthy Eating Index (HEI) assesses dietary quality on whether a set of foods aligns with recommendations based on the Dietary Guidelines for Americans (DGAs) rather than on actual intake of food and nutrients [15]. Similarly, both the DASH diet score and the Mediterranean Diet score (MedDiet score) assess dietary intake based on previously established food and nutrient components to measure adherence to the DASH diet and the Mediterranean diet, respectively [16,17]. To address these limitations, Shivappa et al. (2014) developed the Dietary Inflammatory Index (DII) to assess the overall inflammatory potential of the diet [3]. This new approach includes pro- and anti-inflammatory dietary components simultaneously in the index, which takes into consideration the synergistic or antagonistic effects of nutrients and bioactive compounds when consumed together [6,7,18]. DII was created based on the previous literature that had examined the associations of dietary components with six inflammatory biomarkers (i.e., IL-1β, IL-4, IL-6, IL-10, TNF-α, and CRP).

DII was first validated among a population of healthy Americans (mean age: 49 years), participating in the SEASONS study [19]. Dietary data were derived from 24 h dietary recalls and 7-day dietary recall, and the authors used high-sensitivity CRP (hs-CRP) as the construct validator. DII was also validated among a sample of Japanese individuals (mean age: 57 years) using a food frequency questionnaire (FFQ), dietary records, and hs-CRP and IL-6 [20]. The construct validation of DII was carried out in a population of African Americans (mean age: 54.8 ± 11.4 years) using CRP only, and in postmenopausal women using FFQ-derived DII and IL-6, hs-CRP, and the tumor necrosis factor alpha receptor 2 [21,22]. None of the above studies used a comprehensive panel of inflammatory biomarkers in their analyses.

Several studies to date have examined the association of DII with various health conditions and measures in young adults, including depression, stress and anxiety, sleep quality, glycemic index, glucose regulation, insulin resistance and metabolic syndrome, and bone health and body composition [23,24,25,26,27,28,29,30]. Some of these studies also measured CRP levels. We only found one study with the primary objective of comparing plant-based dietary indexes and the DII with hs-CRP among female college students. The authors found that there was a moderate and positive correlation between hs-CRP and energy-adjusted DII (r = 0.46) among women aged 19–35 years in Saudi Arabia [31]. However, no study to date has been conducted with the sole purpose of validating the DII among young adult women in the United States or any other country, or with a panel of inflammatory biomarkers aside from CRP. CRP is a nonspecific biomarker for systemic inflammation and a surrogate marker for cytokines, such as IL-6 and IL-1β [32,33,34]. CRP is accepted as a predictor of low-grade inflammation and chronic diseases, especially in older adults [5], but may be an inappropriate measure of inflammation in younger adults.

Our objective in this study was to conduct a construct validation of DII, using a food frequency questionnaire (FFQ)-derived DII, a robust panel of 14 inflammatory biomarkers, and an overall inflammatory score derived from these biomarkers, in age-adjusted and multiple adjusted regression models. Our population comprised college-aged women in the United States, who were participants of the University of Massachusetts Vitamin D Study (UMVDS). We hypothesized that a more inflammatory diet (i.e., higher DII score) would be positively associated with pro-inflammatory cytokines (e.g., IL-1β, IL-6, TNF-α), and negatively associated with anti-inflammatory cytokines (e.g., IL-4, IL-10, IL-13) [35].

## 2. Materials and Methods

### 2.1. Dataset and Study Sample

We conducted a cross-sectional secondary data analysis of the UMVDS. Participants were 393 healthy, college-aged women (18–31 years). Data were collected between 2006 and 2014. The original study protocol was approved by the Institutional Review Board at the University of Massachusetts, and all participants provided informed consent before data collection. The protocol, details of data collection, and the original inclusion and exclusion criteria for the UMVDS have been described previously [36]. Briefly, women of reproductive age were eligible if they were currently menstruating and were not taking corticosteroids, anabolic steroids, anticonvulsants, cimetidine, or propranolol. Furthermore, women were not eligible if they reported a history of hypertension, elevated cholesterol, kidney or liver disease, bone disease (e.g., osteomalacia), digestive disorders, rheumatologic disease, multiple sclerosis, thyroid disease, hyperparathyroidism, cancer, type 1 or type 2 diabetes, or polycystic ovarian syndrome [37]. Participants completed questionnaires on demographic, behavioral, dietary, and medical factors. Questionnaires and the study measurements, including blood draws, were completed in a single clinic visit during the mid-luteal phase of the participant’s cycle (i.e., 5–7 days before the start date of their next menses). The blood samples were processed within 2 h of blood draw and stored at −80 °C.

For the purposes of this study, we only included those who had data on all cytokines (*N* = 277) and CRP (*N* = 283). We excluded those who had CRP levels above 10 mg/L (*N* = 38), as this is an indication for acute infection or medication use [38]. We also excluded participants with missing data on our covariates, such as race (*N* = 1), current smoking status (*N* = 57), and physical activity (*N* = 4). All participants had completed the FFQ. We excluded participants who had implausibly low (i.e., <600 Kcal/d) (*N* = 4) or implausibly high (i.e., >5000 Kcal/d) (*N* = 4) caloric intake per day, as reported by the FFQ. There were no participants with implausibly low (i.e., BMI < 15 kg/m^2^) or implausibly high BMI (i.e., BMI > 50 kg/m^2^) in our dataset. The final number of participants included in our analyses were 242 for CRP and 267 for the 13 cytokines. 

### 2.2. Diet

Participants’ dietary intake was assessed using a slightly modified version of the 131-item Harvard Food Frequency Questionnaire (FFQ), which has been previously validated for use in US women [39]. Participants reported their intake over the previous two months.

We utilized DII to assess the inflammatory potential of the diet. DII is composed of 45 items (36 nutrients and nine foods), including macronutrients, micronutrients, and flavonoids. The higher the DII score, the more pro-inflammatory the diet is, and the more negative the score, the more anti-inflammatory the participant’s diet is. The details of DII development and calculation have been published previously [3]. 

Using the Harvard FFQ in this study, 29 of the 45 DII diet components were available for the calculation of a DII score. These dietary components included: energy, carbohydrate, protein, total fat, saturated fat, monounsaturated fatty acid, polyunsaturated fatty acid, omega-3, omega-6, trans fat, cholesterol, alcohol, caffeine, fiber, iron, magnesium, zinc, selenium, niacin, thiamin, riboflavin, folic acid, beta-carotene, and vitamins B6, B12, A, C, D, and E. DII items that were not available for analysis included eugenol, garlic, ginger, onion, saffron, turmeric, green/black tea, flavan-3-ol, flavones, flavonols, flavonones, anthocyanidins, isoflavones, pepper, thyme/oregano, and rosemary. We categorized DII scores into quartiles (Q1, Q2, Q3, Q4), in order to minimize non-differential misclassifications of exposure.

### 2.3. Inflammation

Serum cytokine levels were examined using a microsphere-based suspension microarray (Assay Gate, Ijamsville, MD, USA), and measured using a Bio-Plex 200 Bead Reader System (Bio-Rad, Hercules, CA, USA). Inflammatory biomarkers assayed included IL-1β, IL-2, IL-4, IL-5, IL-6, IL-7, IL-8, IL-10, IL-12 (p70 subunit), IL-13, TNF-α, granulocyte macrophage colony stimulating factor (GMCSF), and interferon gamma (IFN-γ). Laboratory methods for multiplexed approach based on an enzyme-linked immunosorbent assay have been described previously [40,41]. Samples were run in duplicate with internal standards. Coefficients of variation for each inflammatory biomarker were less than 10%. CRP was assayed at the University of Massachusetts Amherst. The CV for CRP was 4.1%. All the inflammatory biomarkers in our sample were heavily right-skewed. Therefore, we log-transformed all the inflammatory biomarkers, before entering them in the regression models, and then back-transformed the results for interpretation and reporting. 

### 2.4. Covariates Assessment

The covariate information was collected through questionnaires (e.g., age, race/ethnicity, smoking status, physical activity levels (PA)), or measured directly during the clinic visit (e.g., weight, height, waist circumference (WC)). In the regression models, we adjusted for variables that were associated with our dependent variables (i.e., inflammatory biomarkers) or were confounders in the association of DII with these biomarkers. These variables included age, race/ethnicity (dichotomized into White or Other), BMI (dichotomized into BMI < 25 kg/m^2^ and BMI ≥ 25 kg/m^2^), WC (dichotomized into <35 or ≥35 inches based on disease risk for non-pregnant women (CDC)), PA, smoking status (dichotomized into current smoker or never/past smoker), and current nonsteroidal anti-inflammatory drug (NSAID) use, such as ibuprofen/Advil/Motrin, Naproxen/Aleve, Indomethacin (dichotomized into currently using and not currently using). The compendium of physical activities was used in UMVDS to assess the energy expenditure of PA. The compendium was developed by Ainsworth et al. (1993) for the Nurses’ Health Study II, validated previously, and updated twice in 2000 and 2011 [42,43]. This compendium determines the amount of time spent each week in walking, jogging, running, bicycling, aerobics/dancing, tennis/racket sports, swimming, yoga/Pilates, and weight training. Response options included ranges from zero minutes to 11+ h per week. Then, metabolic equivalent task (MET) hours per week of PA were calculated based on the intensity of each activity: light intensity (1.6–2.9 METs), moderate intensity (3–5.9 METs), and vigorous intensity (≥6 METs) [44]. 

### 2.5. Statistical Analysis

We used scatter plots to examine the linearity of associations between covariates and the exposure (i.e., DII) and outcomes (i.e., inflammatory biomarkers). The distributions of all inflammatory biomarkers were right-skewed. We performed log transformation to normalize the distributions. We utilized pairwise correlations and variance inflation factors to test for multicollinearity between variables. One-way analysis of variance (ANOVA) was used for the distribution of continuous variables (i.e., age, physical activity, cytokines) across the quartiles of DII, and the Chi square test was used for the categorical covariates (i.e., race, BMI, WC, smoking status, NSAID use).

Inflammatory biomarkers were entered in the regression models as continuous variables and DII was used in quartiles as a categorical variable. Linear regression models were used to assess the association of DII quartiles with inflammatory biomarkers. We ran likelihood ratio tests, using all the covariates that are included in the fully adjusted model, to find the most parsimonious model for each outcome measure (i.e., each inflammatory biomarker).

We built three regression models for each inflammatory biomarker: (1) an age-adjusted model (Model 1); (2) the most parsimonious model based on the results of the likelihood ratio test, adjusted for BMI and NSAID use (Model 2); and (3) a full model adjusted for all covariates (Model 3). In reporting the results, we back-transformed the results of the regression models by exponentiating the coefficients and values. Therefore, the referent value for the regression models was 1 (exp (β = 0) = 1) rather than 0 (β = 0). A *p* < 0.05 was considered significant.

All data analyses were conducted using Stata Statistical Software (StataCorp. 2017. Stata Statistical Software: Release 16. College Station, TX, USA: StataCorp LLC).

## 3. Results

The characteristics of the participants are presented in Table 1, including the mean score of DII in each of its quartiles. The mean age of the population was 21.4 ± 2.9 years, and mean BMI was 23.0 ± 3.2 kg/m^2^. There were no significant differences across DII quartiles in terms of characteristics and covariates, except for age and PA *(p* < 0.05). Those in DII Q4 had the lowest level of PA compared to Q1. DII ranged between −4.80 and +4.74 among 267 participants, with a mean of 0.06 ± 2.08.

The univariate analysis results of the inflammatory biomarkers, including the mean, median, and interquartile range (IQR), are presented in Table 2.

The result of the linear regression models assessing the linear relation of DII and the back-transformed CRP (*N* = 242) and all other inflammatory biomarkers (*N* = 267) in Models 1, 2, and 3 are presented in Table 3. We did not find significant associations between DII and any of the inflammatory biomarkers, except for IL-10, which is an anti-inflammatory cytokine. In the back-transformed data in Model 1, IL-10 levels were lower in Q2, Q3, and Q4 compared to Q1, our referent (Q4 vs. Q1 β: 0.63; 95% CI: 0.43, 0.93; *p*-trend = 0.04). Results remained the same in Models 2 and 3 (Q4 vs. Q1 β: 0.63; 95% CI: 0.43, 0.92; *p*-trend = 0.03; and β: 0.62; 95% CI: 0.42, 0.93; *p*-trend = 0.04, respectively). The decrease in IL-10 levels was not linear and we did not find a dose–response association between DII and IL-10. We also did not find the same significant association in the multivariate-adjusted model for Q2 (β = 0.91; 95% CI 0.62, 1.33), or Q3 (β = 0.96; 95% CI 0.66, 1.39).

## 4. Discussion

This is the first study to assess the association of DII and a robust panel of inflammatory biomarkers among a population of exclusively young adult women. While previous research has assessed DII scores against inflammatory biomarkers in the adult population, no study has done so solely in a young adult population (aged 18–30 years). Our analysis did not support the existence of an association between DII and inflammatory biomarkers in any of the models. The only significant and negative association found was between DII and IL-10 in all three models. In other words, compared with an anti-inflammatory diet, a more pro-inflammatory diet was associated with lower levels of IL-10, an anti-inflammatory cytokine, which partly aligns with our hypothesis.

Compared with studies assessing DII against biomarkers in a general adult population, the overall non-significance found in this analysis is contradictory. For example, DII was initially validated among an older population (mean age: 49 ± 12 years), with only six out of the 14 inflammatory markers we assessed utilized in its development (i.e., IL-1β, IL-4, IL-6, IL-10, TNF-α, and CRP) [3]. Also, previous analyses that have assessed DII against CRP, IL-6, IL-4, and TNF-α in older populations compared to ours, have found significant associations with DII scores [19,20,21,22]. This contradiction with our results may be due in part to overall systemic inflammation increasing with age. Inflammation is mainly considered a condition of older age, which occurs due to the dysregulation of the normal immune response of the body, resulting in the development of chronic inflammation, mediated by dysregulated cytokines and chemokines [45]. In fact, previous studies have confirmed this age-related association with pro-inflammatory biomarkers, particularly CRP and IL-6 [46,47,48]. Inflammation may be present in younger individuals. However, it is often due to a health condition or disease state [49]. Generally, healthy young adult populations, like the one used in this analysis, have relatively lower systemic inflammation compared to older populations [49]. Therefore, we may not have had sufficient variation in terms of inflammation in our dataset to compare the impact of DII on different levels of inflammation, considering that our population was young (mean age: 21.4 years) and predominantly healthy (e.g., normal BMI and WC, non-smokers). The relatively lower levels of circulating pro-inflammatory biomarkers in young adults may explain the lack of significant associations with DII. This could also explain why the only significant result of the current analysis was with IL-10, an anti-inflammatory biomarker. While pro-inflammatory biomarkers increase with age, anti-inflammatory biomarkers tend to decrease with age [50,51]. With lower levels of systemic inflammation and higher concentrations of IL-10 found in the greater young adult healthy population, this sample of generally healthy young females may further substantiate the significant result observed between DII scores and IL-10.

We found that higher DII scores (i.e., a more pro-inflammatory diet) are associated with lower IL-10 (i.e., anti-inflammatory cytokine). This may be explained by previous research showing that women typically consume less calories across all age groups, which results in their higher DII scores (i.e., more pro-inflammatory), even if they are not following an unhealthy dietary pattern, such as the Western-type diet [52]. This is due to the fact that lower caloric consumption is associated with consumption of lower amounts of nutrients that may be anti-inflammatory, which results in higher DII scores. As nutrient intake is positively correlated with energy intake, an increase or decrease in caloric intake can impact non-energy adjusted DII scores [53,54]. Specifically, in this sample of young adult women (*N* = 272), the mean caloric intake was 1478 ± 487 in DII Q4 (*N* = 96) and 2976 ± 1045 in DII Q1 (*N* = 98). This means that in this sample of healthy young adult women, higher calorie consumption led to a more anti-inflammatory diet and increased levels of IL-10, an anti-inflammatory biomarker. Therefore, college-aged women have healthier inflammatory outcomes when consuming more calories those who consume less calories, as demonstrated by our data. Additionally, this highlights that the consideration of energy-adjustment should be taken when assessing dietary patterns among women.

This was the first study to assess the association of DII and inflammatory biomarkers among a population of young adult women. This is also the only study to date that has examined the correlation of DII with inflammation utilizing a panel of 14 inflammatory biomarkers. Our study was not without limitations. This was a cross-sectional study; hence, we cannot infer causality in the association of DII and inflammation. Furthermore, we had a small sample size, which may have resulted in our predominantly null findings. The previous articles validating or performing construct validation of DII, which had found significant results, had population sizes of at least twice the number we had in our analysis [19,20,22]. Our population consisted of mainly White, young, healthy adults, most of whom had healthy lifestyle habits, such as being physically active. As a result, most of the population had low inflammation levels, which reduced the contrast in comparing inflammation levels. This also limits the generalizability of our research to people of other races and ethnicities, as well as young adults with other lifestyle habits and health status. Finally, only 29 out of the 45 components of DII were available for analysis. However, this is comparable to previous studies utilizing DII, as most FFQs and most other commonly used dietary assessments tools generate a limited number of components of DII [55]. The creators of DII also report that using a more limited number of DII components in their validation study did not attenuate the association between DII and inflammatory biomarkers (e.g., CRP) [55]. 

## 5. Conclusions

DII may not be a suitable tool in assessing the inflammatory potential of diet among young women, as it was validated among an older population, utilizing a limited number of inflammatory biomarkers. Other methods of dietary assessment may be used in evaluating the inflammatory potential of diet among young adults, or caloric intake should be separately considered when utilizing DII. Future researchers may examine this correlation in a prospective study and among larger and more diverse populations.

## Figures and Tables

**Table 1:** Participant characteristics by Dietary Inflammatory Index (DII) quartiles among 267 participants from the UMass Vitamin D Study (UMVDS) 2006–2014.

		DII Quartiles				
N (%)		Q1 67 (25.1)	Q2 66 (24.7)	Q3 72 (27.0)	Q4 62 (23.2)	
DII scores, mean (SD)		−2.6 (0.8)	−0.7 (0.5)	1.0 (0.50)	2.7 (0.7)	
Characteristics		Mean (SD)	Mean (SD)	Mean (SD)	Mean (SD)	*p*-Value
Age (y)		21.1 (2.9)	22.2 (3.6)	20.9 (2.3)	21.2 (2.7)	0.005 *
Physical Activity (PA) (MET hours)		80.1 (58.3)	56.6 (51.6)	51.2 (44.0)	34.4 (35.5)	0.001 *
Body Mass Index (kg/m^2^)		22.6 (2.7)	23.0 (3.3)	23.4 (3.4)	23.1 (3.3)	0.25
Waist Circumference (cm)		75.6 (8.2)	78.0 (8.3)	78.2 (8.9)	78.2 (9.5)	0.64
Subject Characteristics		N (%)	N (%)	N (%)	N (%)	
Race	White	59 (88.0)	57 (86.3)	68 (94.5)	53 (85.5)	0.33
	Other	8 (12.0)	9 (13.7)	4 (5.5)	9 (14.5)	
Smoking	Never/Past Smoker	64 (95.5)	62 (93.9)	66 (91.7)	60 (96.8)	0.60
	Current Smoker	3 (4.5)	4 (6.1)	6 (8.3)	2 (3.2)	
BMI	Underweight or Normal Weight	54 (80.6)	52 (78.8)	53 (73.6)	47 (75.8)	0.77
	Overweight or Obese	13 (19.4)	14 (21.2)	19 (26.4)	15 (24.2)	
Waist Circumference	Low Risk (≤35″)	60 (89.5)	59 (89.4)	62 (86.1)	53 (85.5)	0.84
	High Risk (>35″)	7 (10.5)	7 (10.6)	10 (13.9)	19 (14.5)	
NSAID Use	Currently Using	11 (16.4)	19 (28.8)	23 (32.0)	21 (33.9)	0.10
	Not Currently Using	56 (83.6)	47 (71.2)	49 (68.0)	41 (66.1)	

* Results were statistically significant (*p* < 0.05).

**Table 2:** Univariate statistics of inflammatory biomarkers among 267 participants from the UMass Vitamin D Study (UMVDS) 2006–2014.

Inflammatory Biomarkers	Mean (SD)	Median	IQR ^a^	Min	Max
CRP (mg/L) ^b^	2.6 (2.0)	1.9	1.1, 3.6	0.3	8.9
Interleukin (IL)-1β ^c^	1.8 (3.8)	0.7	0.1, 2.4	0.1	48.2
IL-2	7.2 (59.2)	1.5	0.6, 2.8	0.1	948.8
IL-4	22.8 (166.6)	5.6	3.1, 9.5	0.1	2581.4
IL-5	0.6 (2.6)	0.2	0.1, 0.4	0.1	41.3
IL-6	7.0 (15.7)	2.2	1.2, 4.2	0.1	111.1
IL-7	3.1 (3.7)	2.1	0.5, 4.2	0.1	35.8
IL-8	2.6 (6.3)	1.7	1.2, 2.3	0.1	91.7
IL-10	32.3 (108.7)	10.5	6.3, 21.8	0.5	1586.6
IL-12p70	41.6 (228.6)	3.5	1.5, 9.8	0.1	2919.9
IL-13	4.6 (15.2)	2.3	0.9, 4.1	0.1	227.5
TNF-α	6.0 (5.6)	4.8	2.5, 8.1	0.5	64.2
GMCSF	15.5 (22.2)	7.0	1.9, 24.1	0.1	238.3
IFN-Υ	8.6 (49.4)	2.3	0.8, 4.6	0.1	620.0

(a) IQR: interquartile range; (b) CRP: C-reactive protein. Data on CRP were available for 242 participants. (c) Unit measurements for cytokines (interleukins, GMCSF, IFN-Υ, and TNF-α) are in pg/mL.

**Table 3:** Back-transformed ^a^ estimated beta coefficients and standard errors for the association of the Dietary Inflammatory Index quartiles and inflammatory biomarkers in unadjusted and multivariable-adjusted linear regression models among 267 participants from the UMass Vitamin D Study (UMVDS) 2006–2014.

		Quartiles of DII			
	Q1	Q2	Q3	Q4	*p*-Trend
C-Reactive Protein (CRP) ^b^					
Model 1: Age-Adjusted, β (SE)	Ref ^e^	0.92 (0.13)	1.02 (0.14)	1.04 (0.15)	0.63
Model 2: Most Parsimonious ^c^, β (SE)	Ref	0.90 (0.12)	0.98 (0.13)	1.00 (0.14)	0.84
Model 3: Fully Adjusted ^d^, β (SE)	Ref	0.88 (0.13)	0.94 (0.13)	0.99 (0.15)	0.97
Interleukin (IL)-1β					
Model 1: Age-Adjusted, β (SE)	Ref	1.51 (0.37)	1.44 (0.35)	1.34 (0.33)	0.25
Model 2: Most Parsimonious ^c^, β (SE)	Ref	1.57 (0.38)	1.57 (0.38)	1.45 (0.36)	0.15
Model 3: Fully Adjusted ^d^, β (SE)	Ref	1.53 (0.39)	1.52 (0.38)	1.35 (0.36)	0.27
IL-2					
Model 1: Age-Adjusted, β (SE)	Ref	1.69 (0.38)	1.74 (0.38)	1.19 (0.27)	0.35
Model 2: Most Parsimonious ^c^, β (SE)	Ref	1.71 (0.38)	1.86 (0.41)	1.26 (0.28)	0.23
Model 3: Fully Adjusted ^d^, β (SE)	Ref	1.64 (0.37)	1.69 (0.38)	1.11 (0.27)	0.57
IL-4					
Model 1: Age-Adjusted, β (SE)	Ref	1.15 (0.23)	1.43 (0.29)	1.12 (0.23)	0.35
Model 2: Most Parsimonious ^c^, β (SE)	Ref	1.19 (0.24)	1.50 (0.30)	1.19 (0.25)	0.22
Model 3: Fully Adjusted ^d^, β (SE)	Ref	1.16 (0.24)	1.42 (0.29)	1.11 (0.25)	0.40
IL-5					
Model 1: Age-Adjusted, β (SE)	Ref	1.11 (0.16)	0.97 (0.14)	1.15 (0.17)	0.55
Model 2: Most Parsimonious ^c^, β (SE)	Ref	1.09 (0.16)	0.99 (0.14)	1.16 (0.18)	0.48
Model 3: Fully Adjusted ^d^, β (SE)	Ref	1.15 (0.17)	1.02 (0.15)	1.19 (0.19)	0.43
IL-6					
Model 1: Age-Adjusted, β (SE)	Ref	1.05 (0.24)	1.28 (0.28)	0.96 (0.22)	0.86
Model 2: Most Parsimonious ^c^, β (SE)	Ref	1.09 (0.25)	1.27 (0.28)	0.97 (0.22)	0.89
Model 3: Fully Adjusted ^d^, β (SE)	Ref	1.02 (0.23)	1.16 (0.27)	0.88 (0.21)	0.78
IL-7					
Model 1: Age-Adjusted, β (SE)	Ref	1.05 (0.24)	1.28 (0.29)	1.06 (0.25)	0.57
Model 2: Most Parsimonious ^c^, β (SE)	Ref	1.10 (0.25)	1.35 (0.31)	1.13 (0.26)	0.42
Model 3: Fully Adjusted ^d^, β (SE)	Ref	(0.25)	1.27 (0.30)	1.06 (0.27)	0.60
IL-8					
Model 1: Age-Adjusted, β (SE)	Ref	1.05 (0.13)	1.05 (0.12)	0.99 (0.12)	0.96
Model 2: Most Parsimonious ^c^, β (SE)	Ref	1.07 (0.13)	1.08 (0.13)	1.03 (0.13)	0.77
Model 3: Fully Adjusted ^d^, β (SE)	Ref	1.10 (0.13)	1.08 (0.13)	1.07 (0.14)	0.60
IL-10					
Model 1: Age-Adjusted, β (SE)	Ref	0.91 (0.17)	0.97 (0.18)	0.63 (0.12)	0.04 *
Model 2: Most Parsimonious ^c^, β (SE)	Ref	0.93 (0.17)	0.95 (0.17)	0.63 (0.12)	0.03 *
Model 3: Fully Adjusted ^d^, β (SE)	Ref	0.91 (0.17)	0.96 (0.18)	0.62 (0.12)	0.04 *
IL-12p70					
Model 1: Age-Adjusted, β (SE)	Ref	1.44 (0.46)	1.82 (0.57)	1.06 (0.34)	0.61
Model 2: Most Parsimonious ^c^, β (SE)	Ref	1.43 (0.46)	1.77 (0.56)	1.04 (0.34)	0.68
Model 3: Fully Adjusted ^d^, β (SE)	Ref	1.43 (0.47)	1.69 (0.55)	1.03 (0.36)	0.75
IL-13					
Model 1: Age-Adjusted, β (SE)	Ref	1.10 (0.26)	1.28 (0.30)	1.18 (0.29)	0.37
Model 2: Most Parsimonious ^c^, β (SE)	Ref	1.11 (0.26)	1.29 (0.30)	1.19 (0.29)	0.36
Model 3: Fully Adjusted ^d^, β (SE)	Ref	1.07 (0.26)	1.17 (0.28)	1.09 (0.28)	0.64
TNF-α					
Model 1: Age-Adjusted, β (SE)	Ref	1.28 (0.16)	1.16 (0.14)	1.20 (0.15)	0.24
Model 2: Most Parsimonious ^c^, β (SE)	Ref	1.29 (0.16)	1.20 (0.15)	1.24 (0.16)	0.15
Model 3: Fully Adjusted ^d^, β (SE)	Ref	1.32 (0.17)	1.21 (0.16)	1.26 (0.17)	0.17
GMCSF					
Model 1: Age-Adjusted, β (SE)	Ref	1.53 (0.42)	1.48 (0.39)	1.39 (0.38)	0.24
Model 2: Most Parsimonious ^c^, β (SE)	Ref	1.57 (0.42)	1.63 (0.43)	1.51 (0.41)	0.12
Model 3: Fully Adjusted ^d^, β (SE)	Ref	1.55 (0.43)	1.55 (0.43)	1.43 (0.42)	0.23
IFN-Υ					
Model 1: Age-Adjusted, β (SE)	Ref	1.31 (0.33)	1.36 (0.33)	1.04 (0.26)	0.78
Model 2: Most Parsimonious ^c^, β (SE)	Ref	1.37 (0.34)	1.45 (0.35)	1.11 (0.28)	0.58
Model 3: Fully Adjusted ^d^, β (SE)	Ref	1.38 (0.35)	1.42 (0.35)	1.13 (0.30)	0.60

(a) Log transformation: ln(Y) = β0 + β1 × X1 + β2 × X2 + … + βN × XN. Back-transformation: Y = exp (β0 + β1 × X1 + β2 × X2 + … + βN × XN); (b) CRP: C-reactive protein. Data on CRP were available for 242 participants; (c) Model 2: adjusted for BMI and NSAID use; (d) Model 3: adjusted for age (years), race (White, non-White), BMI (dichotomized by BMI of 24.9 kg/m^2^), waist circumference (WC) (dichotomized by WC of 35 inches), physical activity (MET-hours per week), smoking status (current smoker, former smoker, and never smoker), NSAID use (currently using, not currently using); (e) This is the back-transformed referent, where β = 0 has been exponentiated. Therefore, the referent is β = 1. (*) Results were statistically significant (*p* < 0.05).

## Data Availability

Data sharing not applicable.
